# Bio-inspired interlocking random 3-D structures for tactile and thermal sensing

**DOI:** 10.1038/s41598-017-05743-w

**Published:** 2017-07-19

**Authors:** Long Pu, Rohit Saraf, Vivek Maheshwari

**Affiliations:** Department of Chemistry, Waterloo Institute of Nanotechnology, University of Waterloo, Waterloo, ON N2L 3G1 Canada

## Abstract

Hierarchical nanostructures are tailored and used routinely in nature to accomplish tasks with high performance. Their formation in nature is accomplished without the use of any patterning process. Inspired by the performance of such structures, we have combined 2-D nanosheets with 1-D nanorods for functioning as electronic skin. These structures made in high density without any patterning process can be easily assembled over large areas. They can sense pressures as low as 0.4 Pa, with a response time in milliseconds. Further, these structures can also detect temperature changes with a non-linear response in the 298–400 K range, which is similar to skins perception of thermal stimuli. We illustrate this effect by showing that the device can differentiate between two 10 µl water droplets which are at room temperature and 323 K respectively.

## Introduction

Hierarchical structures with mechanical and functional integration of distinct nanomaterials and (or) morphologies in a cohesive manner show many remarkable features and properties^1–5^. As a result, such structures made with multiple micron and nano scale components are being actively researched for use in diverse devices ranging from high surface area current collectors in batteries^6–8^, solar cells^9, 10^ and supercapacitors^11, 12^, to biomimetic adhesives^13–15^ and as surface coatings^16–18^. In nature such hierarchical structures, made without the use of any lithography or pattering processes, are ubiquitously present and are used to accomplish specific tasks with great efficiency and unparalleled performance^19–21^. The ongoing expansion of robotic and humanoid platforms requires the development of sensory interfaces such as electronic skin (or tactile sensors), with performance on par with human analogues^22–24^. This will also impact other areas of tactile sensing application such as wearable sensors and medical devices^25–29^. Electronic skin (or tactile) devices that sense pressure have been made using hair like patterned polymer nanofibers^30, 31^, CNT and graphene networks^32–36^, hollow sphere microstructures^37^ and ZnO rods on patterned PDMS micro pillars^38^, among others. Structural interlocking and multi contact conductive pathways that lead to inherent piezo-resistive and percolation effects are the primary basis of pressure sensing in these devices^31, 39^. Being a large area device (human skin has an area of ~2 m^2^, and specifically the palm and fingers have an area of ~120 cm^2^), the translation of non-patterned hierarchical structures as tactile sensors (or electronic skin) will lead to significant benefits due to ease of fabrication and integration. This has to be coupled with high sensitivity and rapid response time for sensing of pressure and a robust integrity^30, 40–42^. Besides pressure, integrated temperature sensing will significantly enhance the capabilities of such electronic skin devices bringing them closer to the multi-functional capabilities of human skin^43–46^. By using a rationally designed hierarchical structure we address these requirements as reported below.

The electronic skin sensor is made by rationally combining 1-D ZnO nanorods (length scale 400 nm) onto 2-D ZnO sheets (length scale of micron) as a hierarchical structure, using simple electrochemical synthesis and without any patterning process. This sensor has, exceptional sensitivity (greater than 10^5^ times change in resistance) in the low-pressure regime, a minimum pressure sensing threshold of at least 0.4 Pa with a response time of less than 2 ms and also the ability to sense temperature. We show that the sensor is able to differentiate between 20 µl and 40 µl water droplets and also differentiate between 10 µl droplets of room temperature and 50 °C. The temperature and pressure stimuli can be differentiated based on their response time scales, which are in seconds and milliseconds respectively. By comparing the response characteristics of sensors with different structuring elements, the exceptional performance of the hierarchical sensor is attributed to its inter-locking structure and the formation of multi-contact pathways due to the rational combination of nanorods with sheets. The number of pathways increases rapidly with pressure leading to the pressure sensing ability. Further, the hierarchical template is used as a pseudocapacitor by conformal electrochemical deposition of a MnO_2_ layer, and compared to planar counter parts it shows an order of magnitude improvement in energy density. This versatility of the hierarchical structure will be advantageous for application in wearable electronic devices, as a single fabrication process can be used for making both a sensor and an energy storage system.

## Results and Discussion

**Figure 1:**
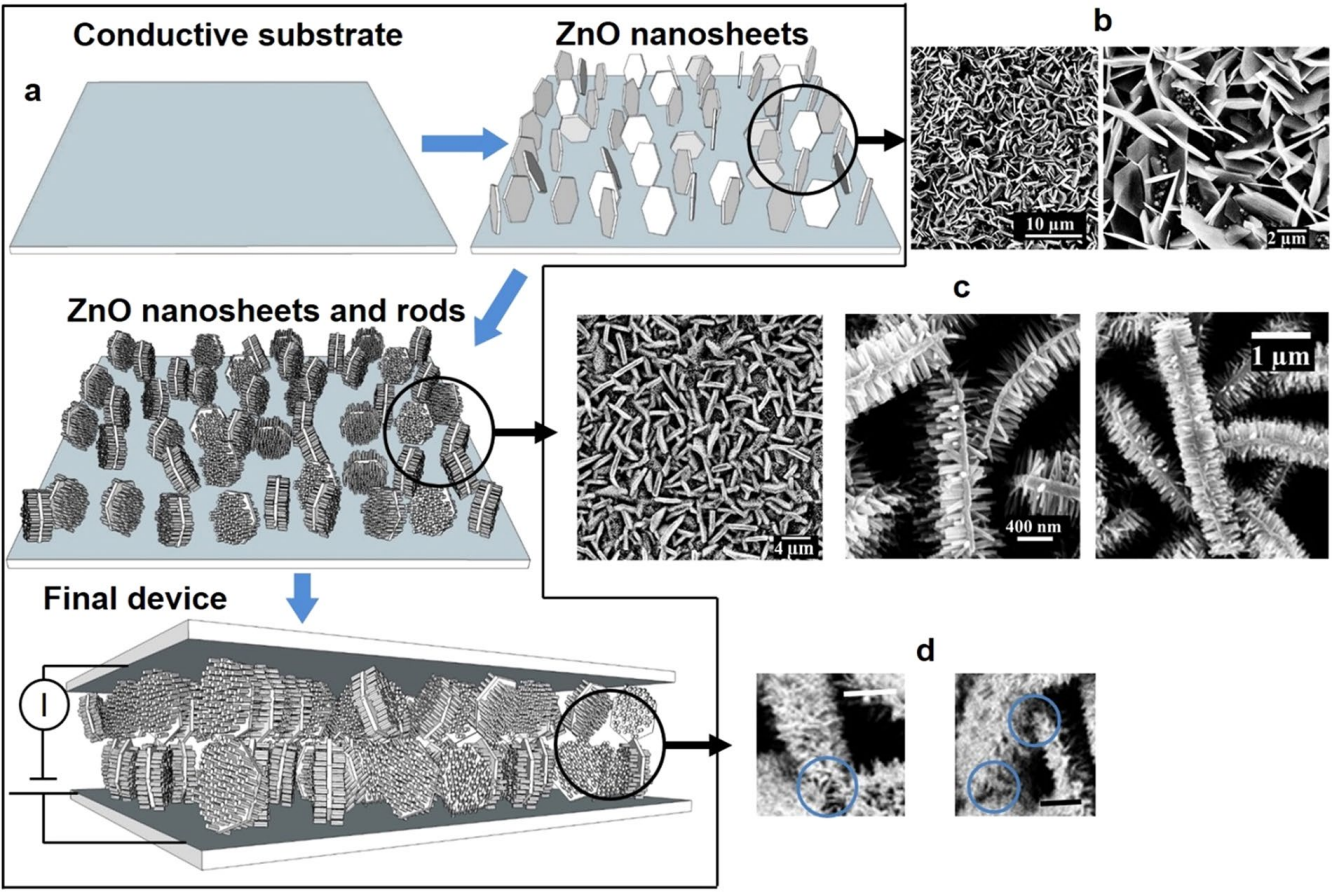
(**a**) Schematic showing the formation of the device structure. (**b**) FESEM images showing the ZnO sheets that form the first layer of the device. The higher resolution image on the right shows that the sheets are microns in size with a thickness on the scale of 50–200 nm. (**c**) FESEM images of the sheet-rod nanostructure. The image on the left shows the high density of the structure. The higher resolution images on right clearly show the sheet outline and the rods growing primarily on the larger area plane of the sheets. The length of the rods is 200–400 nm. (**d**) The cross section of the final device structure shows the interlocking between the sheet-rod nanostructure of the top and bottom electrode. This has been highlighted in blue. The scale bars are 1 µm.

The schematic of the sensor is presented in Fig. 1a. The sensor is made by first forming 2-D ZnO nanosheets (Fig. 1b) on a planar conductive substrate, followed by formation of ZnO nanorods (Fig. 1c). Both morphologies of ZnO are made electrochemically and are controlled by varying the composition of the electrolyte solution^47, 48^. The 1-D rods grow predominately on the large surface area side of the sheets due to its lower resistance and crystal orientation, as can be seen in the field emission scanning electron microscopy (FESEM) images of Fig. 1c and X-ray diffraction data (XRD details in SI). The final ZnO sheet-rod nanostructure is a dense array of these 2-D-1-D units. Two such substrates are placed together and sealed with PDMS. A cross section of the device showing the interlocking contacts is presented in Fig. 1d.

**Figure 2:**
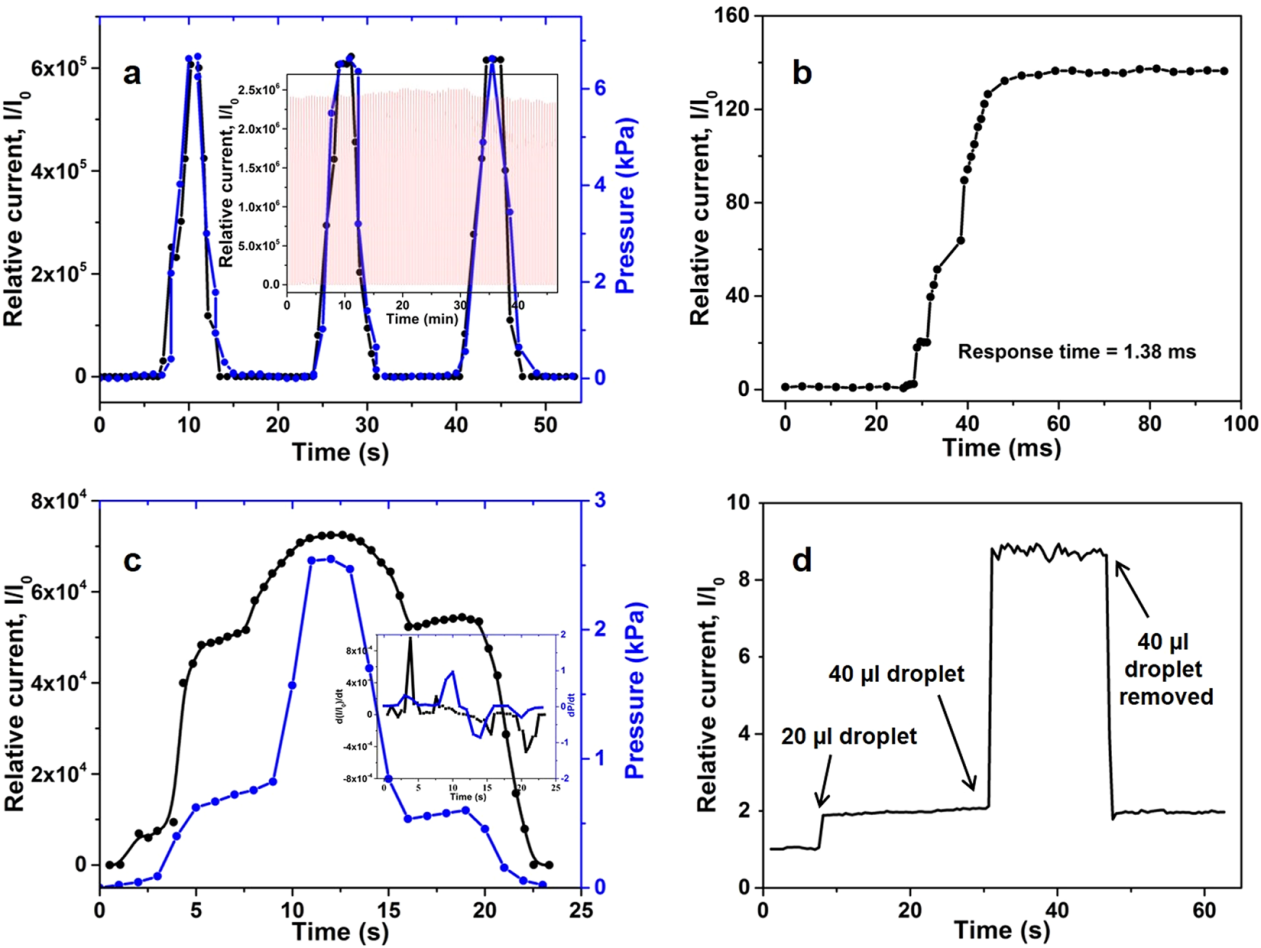
Tactile sensing characteristics of the device. (**a**) The relative current in the sensor increases more than 5 orders of magnitude on increasing the base pressure to ~6.5 kPa. The response from the device is robust as seen in the inset, where over 100 pressure cycles of 10 kPa are applied. (**b**) The device responds in less than 2 ms on being subjected to a 0.01 kPa step increase in load. (**c**) The device responds in step to both positive and negative changes in the pressure stimuli. The inset shows that the derivative of the response tracks that of the applied stimuli. (**d**) The device is sensitive to pressure changes due to placing of small water droplets. Both the initial placement of the droplet and subsequently another one were detected. The device also tracks the removal of one of the droplets.

The response of the device to typical pressure cycles is shown in Fig. 2a and an over 10^5^ order change in current is observed on increasing the pressure to 6.5 kPa. The response is consistent over repeated fast pressure cycling (100 cycles of 10 kPa) as observed in the inset of Fig. 2a. The response time of the sensor is measured as 1.38 ms, on subjecting it to a step change of 10 Pa in pressure that occurs within 20 ms and is illustrated by Fig. 2b. The current accurately tracks the change in pressure, signifying the rapid response from the device. The fast response time in pressure is indicative of the rapid change in the density of conductive pathways in the device. Further as the device structure is made with ZnO this limits viscoelastic effects in the response. The correspondence between the current and pressure is more clearly illustrated in Fig. 2c, the response of the device is in step with the pressure profile tracking both negative and positive derivatives in the pressure-time profile (inset of Fig. 2c). The sensitivity of the device to pressure is illustrated in Fig. 2d, the current changes by 2.2 and 9 times respectively on placing 20 µl (~0.8 Pa) and 40 µl (1.6 Pa) droplets in succession, and it reverts to original level on removing 40 µl the droplet. As illustrated below, the formation of the sheet-rod structure is the key to achieving this performance.

To develop a better understanding of the hierarchical structure, its response is modelled as a power law given by:

1 $${I}_{r}={I}_{0}+A\times {P}^{\alpha }$$

where P is the applied pressure in kPa above the base pressure (arising from the weight of the device), *I*_*r*_ is the relative current, *I*_0_ is the relative current at base pressure (is hence equal to ~1). A is a measure of the conductance of the structures which is affected both by, their inherent conductivity based on composition (in this case ZnO) and their morphology which will affect the change in the density of conduction pathways with pressure. α is dependent on the structure of the template. The product A × α is a measure of the device sensitivity and α also affects the dynamic range of response. A higher α will lead to a greater dynamic range.

**Figure 3:**
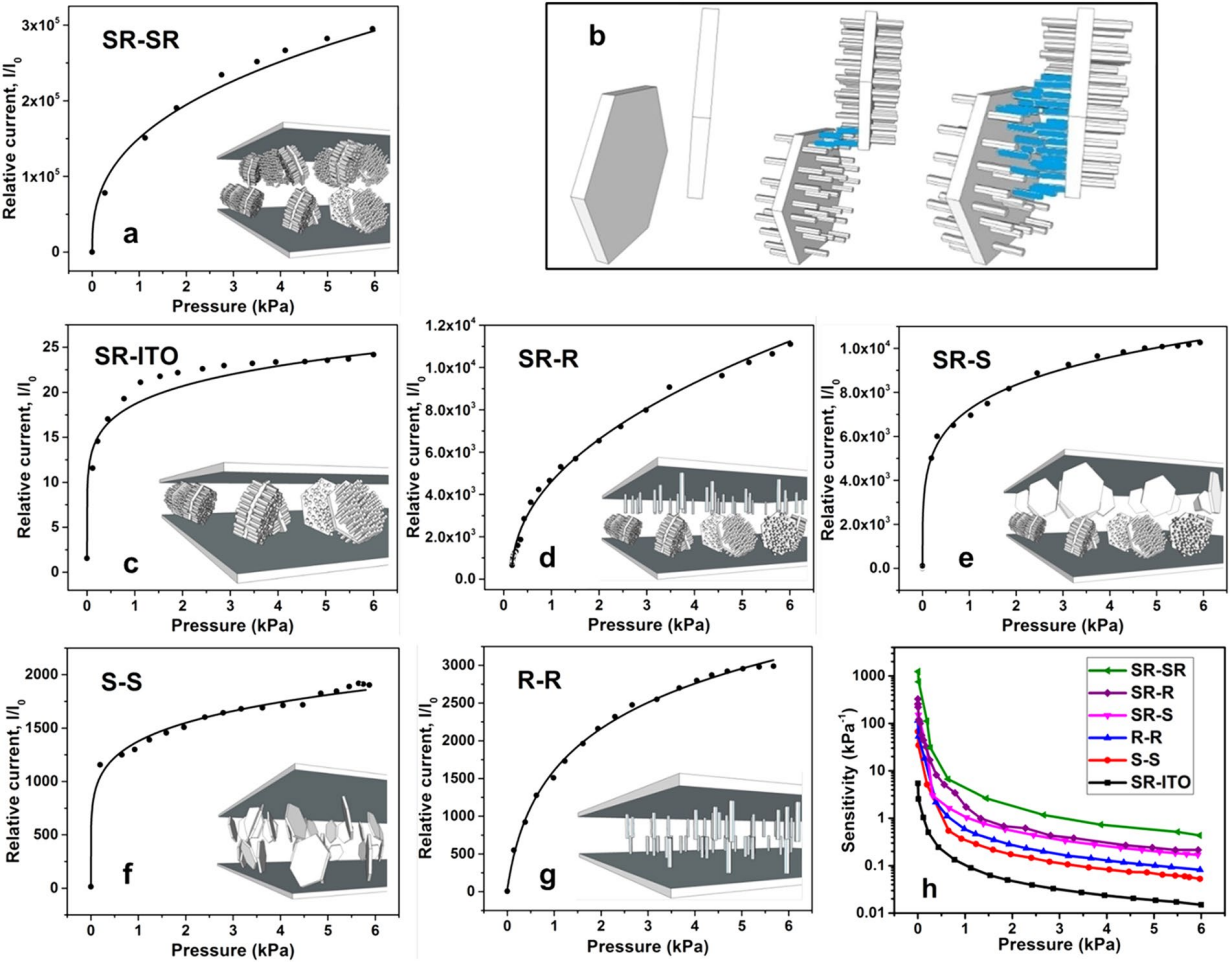
The effect of the structure morphology on the response and sensitivity of the sensor is illustrated for 6 different cases. The response from all the structures can be accurately modelled as a power law function. The lines are the fit from the power law to the data points. (**a**) The SR-SR (hierarchical nanostructure-hierarchical nanostructure) structure has the highest sensitivity due to its hierarchical nature. (**b**) The higher sensitivity in this hierarchical structure results from its greater interaction volume and its variation with pressure compared to just the sheet structure. The response from devices made with the following morphology: (**c**) SR-ITO (hierarchical nanostructure-flat), (**d**) SR-R (hierarchical nanostructure-nanorods), (**e**) SR-S (hierarchical nanostructure-nanosheets), (**f**) S-S (nanosheets-nanosheets) and (**g**) R-R (nanorods-nanorods). (**h**) The sensitivity of all the devices, as calculated from their pressure response, shows a progressive increase with to greater structuring.

We observe that the power law model accurately fits the response from the sheet-rod device as seen in Fig. 3a. The basis for the power law can be described similar to the percolation effects in which the conductance scales with the power of the concentration of the conductive filler (in the insulating matrix), as this increases the conduction pathways^39, 49^. In this tactile sensor, the increase in pressure leads to greater interpenetration (and bending) between the nanostructures which increases the density of the pathways available for electrical conduction. There are two distinct effects in this structured device compared to a flat configuration that result in the observed performance, 1. The structuring by the ZnO sheets provides discreet contact points between the two electrode surfaces. This concentrates the applied pressure and with increasing pressure will lead to a greater overlap between the sheets (both due to interpenetration and bending) making the structure dynamic with pressure. 2. The rods serve to increase the overlap volume for the sheets to make electrical contacts. This is shown in Fig. 3b, with just sheets there has to be direct contact between them for conduction. However in presence of rods (~ 400 nm in length), even sheets separated by 800 nm can serve as conduction channels due to contacts between the rods. Increasing pressure will lead to greater interpenetration and bending of the sheets and rods. This will increase the number of rods in contact between adjacent sheets that serve as conduction pathways. The combined effect of the hierarchical structuring leads to the observed high sensitivity and low detection threshold in the device.

To develop a more comprehensive understanding of the relationship between the device structure and its performance, tactile sensors with different morphologies were characterized and their response was fit to the power law model, Fig. 3c–g. The parameters, A, α, and the sensitivity A × α, (at P = 1) for the samples are reported in Table 1. The complete plots of sensitivity for all the device structures, as calculated from their response are shown in Fig. 3h. Four distinct ranges of behaviors are observed. The device consisting of an Indium tin oxide (ITO) coated flat top electrode with a sheet-rod structure as the bottom electrode has the least sensitivity and the smallest A (~17). The next set of devices consisting of only a single type of structuring on both the electrodes, either nanosheets or rods, show very similar behaviours with an increase in A and sensitivity (by 3–10 times) compared to the flat electrode configuration. The next sets of devices consist of hierarchical structures (nanosheet-rods) on one electrode and a single type of structuring (nanosheets or rods) on the other electrode. The presence of the hierarchically structured electrode further increases the sensitivity (by ~2–8 times) and A (by ~5 times) in these devices. The highest sensitivity and A are achieved with the device that has hierarchically structuring on both the electrodes. The results in A and the sensitivity show that structuring leads to a significant enhancement in tactile sensing due to formation of specific contact points and dynamic modulation of the conduction pathways due to interpenetration and bending of the structures (both sheets and rods). Similar to the ridges on the finger, the presence of structuring will intensify the applied pressure at the contact points, as has been shown with other micro structures^50, 51^. Based on the density of sheets in hierarchical structures, the contact area between the two electrodes is reduced by ~ 85–90% (see supporting information) increasing the pressure experienced by the sheets edges by 7–9 times. A significant improvement also results from the fact that the sheet structures are randomly oriented, as a result when the two substrates are placed in contact only a small fraction of sheet edges will make contact, significantly increasing the effective pressure (see supporting information). The formation of hierarchical structures further improves the device characteristics, as the effective volume for making contacts is increased due to the presence of rods on the sheets. Further in these structures, a single rod on one sheet can also form multi-point contacts (that change with applied pressure) with rods on adjacent sheets, significantly increasing the density of conduction pathways compared to single point contacts.

**Table 1:** The value of the parameters for the power law in equation 1 used for modelling the response from all the different morphologies of the sensors.

Sensing materials	A	α	A × α
3-D ZnO nanostructure and ITO (SR-ITO)	17.67	0.155	2.74
2-D ZnO nanosheets and 2-D ZnO nanosheets (S-S)	1362.35	0.172	234.32
1-D ZnO nanorods and 1-D ZnO nanorods (R-R)	1564.65	0.219	342.66
3-D ZnO nanostructure and 2-D ZnO nanosheets (SR-S)	6970	0.247	1721.60
3-D ZnO nanostructure and 1-D ZnO nanorods (SR-R)	5002	0.459	2295.92
3-D ZnO nanostructure and 3-D ZnO nanostructure (SR-SR)	150545	0.595	89574.28

The model illustrates that increase in the level of structuring leads to higher sensitivity and also improves the dynamic range of the device.

**Figure 4:**
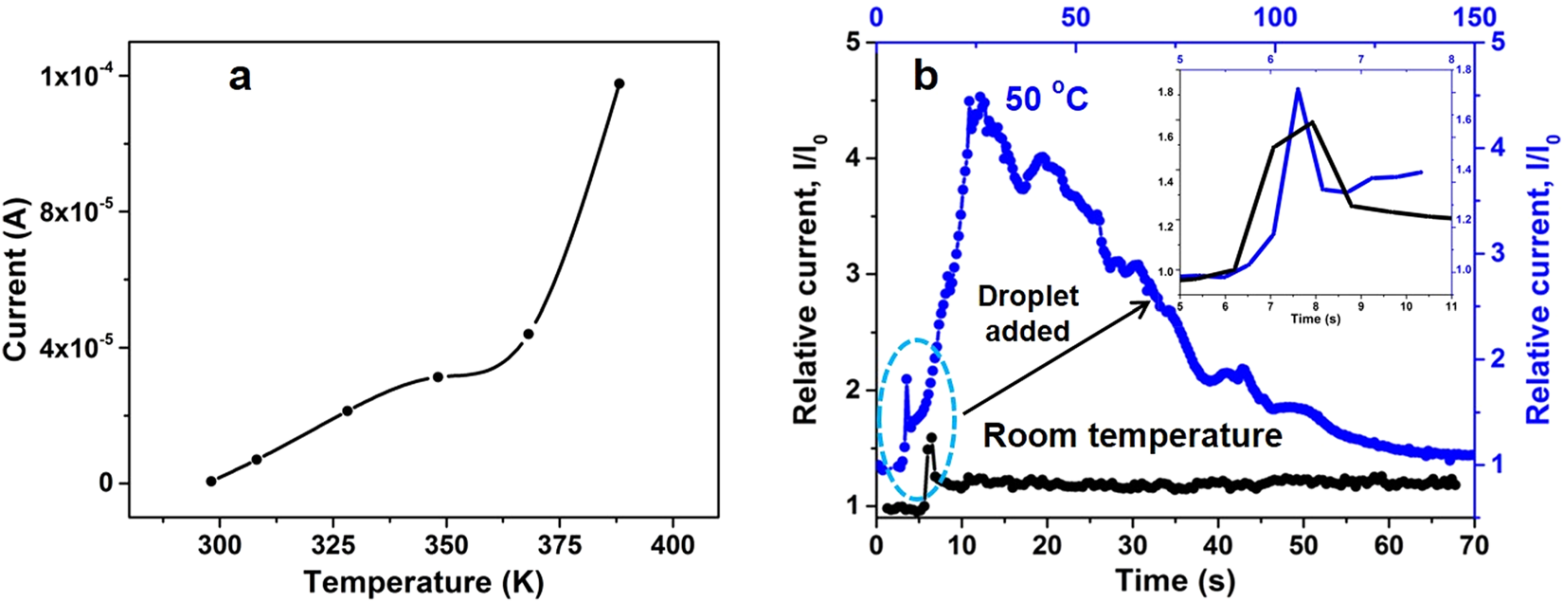
The temperature sensing ability of the device due to its ZnO structure. (**a**) The current across the device at a constant bias of 0.5 V under a base pressure increases monotonically within the temperature range of 298–400 K, as relevant for human skin. (**b**) The device is able to differentiate between two 10 µl size droplets of 298 and 323 K that are dropped on its surface. An initial fast and nearly identical response due to the pressure from the weight of the droplets is observed in both cases. The inset shows a magnified view of this initial response. For the droplet at 323 K a subsequent slow increase in current is observed due to the propagation of the temperature stimuli. After reaching a peak in current a slow decay over ~60 s due to cooling of the droplet is observed.

Combining pressure sensing with the ability to also detect temperature changes will bring electronic skin devices closer to functional capabilities of human skin. Two stimuli can be deciphered in a single sensor based on its differential sensitivity, as has been reported for decoupling shear and normal stresses in tactile sensor^30^. ZnO has a temperature sensitive response in conductivity due to its semiconducting properties and adsorption of gas molecules on surface^52–54^. The basis of temperature sensing is hence based on the material characteristics of the device rather than its hierarchical structure. The human perception of temperature is highly non-linear and is based on the surface being perceived as cool, warm, hot and cold^43, 45^. As a result varying degree of ease, from comfort to slight discomfort and to even pain are perceived due to the magnitude of the temperature stimuli. The response of the hierarchical sensor to temperature is shown in Fig. 4a. A non-linear response is observed and the sensitivity increases with temperature, this will aid the rapid sensing of hot surfaces. Such a response similar to skin is better suited to decipher between varying degrees of temperature stimuli. As seen in Fig. 4b, the sensor is able to differentiate between water droplets (10 µl each) of room temperature and 50 °C. The pressure response to the two droplets is similar and can be observed in the initial spike (within 1 s) that results from dropping of the droplet on the sensor. Following this a short period of increase (~ 4–5s) in current is observed from the 50 °C water droplet due to increase in the temperature, followed by a prolonged period of slow decrease in current, due to the cooling of the droplet to ambient temperature. The sensitivity of the device to pressure is in the range of 1200–0.5 kPa^−1^, while for temperature (298–388 K) it is in the range of 0.016–0.091 K^−1^, this significant difference combined with the difference in response time allows the detection of both pressure and temperature stimuli. The difference in response time is also due to the faster propagation of a stress stimuli compared to a thermal stimuli based on the velocity of elastic waves in solids and the Fourier law of heat conduction.

**Figure 5:**
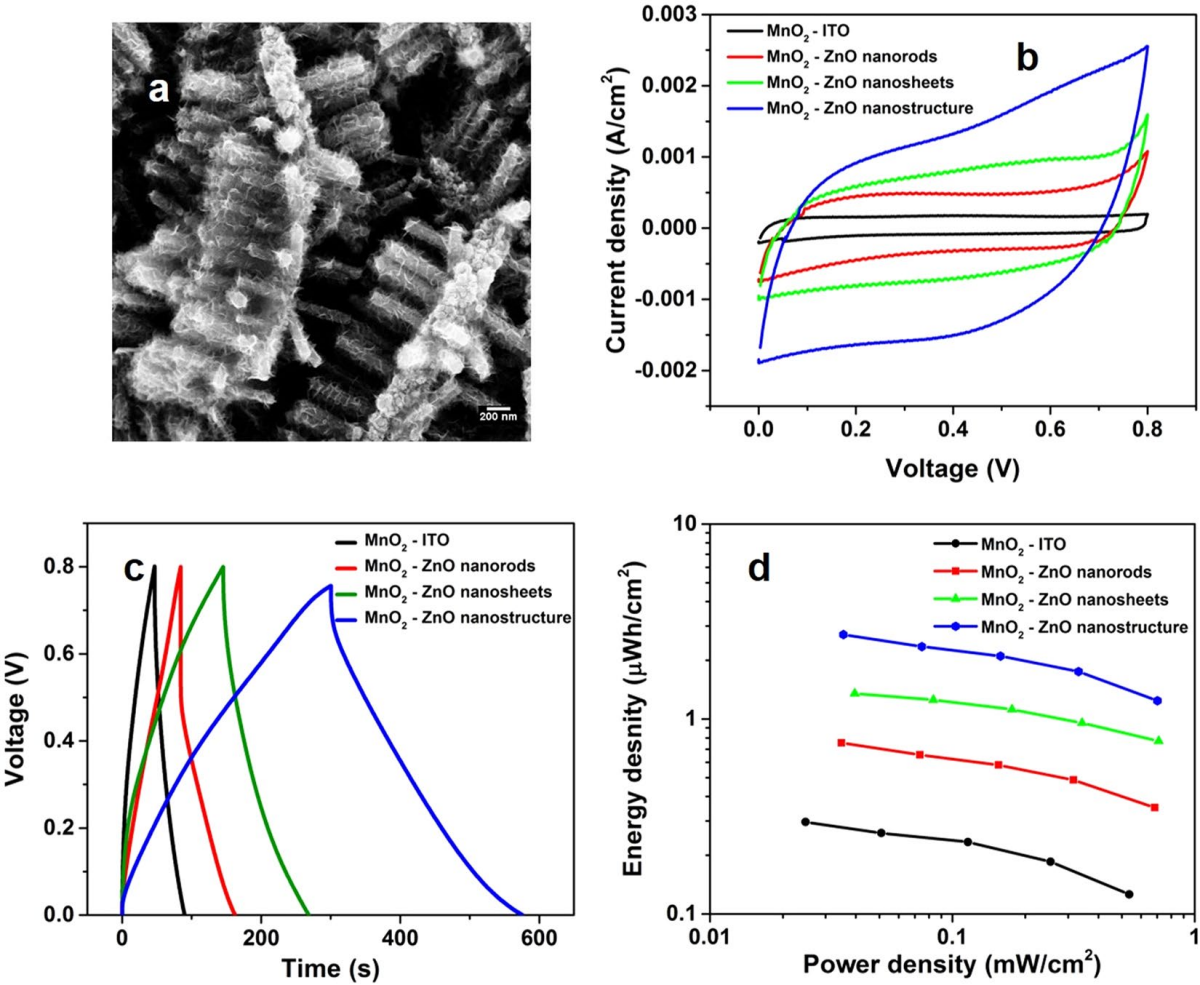
The hierarchical structure leads of a more than one order of magnitude increase in the energy storage capacity of a pseudocapacitor made with MnO_2_, compared to a flat morphology. (**a**) In the FESEM image, the conformal coating of the MnO_2_ on the nanosheet-rod structure is clearly visible. (**b**) CV at 50 mV s^−1^ scan rate, and (**c**) Galvanostatic charge-discharge curves of the different structures showing the effect of morphology. (**d**) The Ragone plots show the better performance of the hierarchical structure in both energy and power density.

The hierarchical sheet-rod structures are also a high surface area template, this effect is utilized for making a pseudo-capacitor by electro-depositing a thin conformal layer of MnO_2_ (FESEM image of Fig. 5a). This pseudo capacitor structure has a pure areal capacitance of 38.7 mF cm^−2^, which is over an order magnitude higher than a similar capacitor made on a flat ITO (Fig. 5b). The structure is not a multi-layer and hence this value is considered as pure areal capacitance. Further if we convert this areal capacitance to volumetric capacitance the values will be highly increased (~100 F cm^−3^), however similar to reporting areal capacitance for multilayer structures this is not an effective approach. The effect of the hierarchical structuring is illustrated in the cyclic voltammetry and galvanostatic charge discharge curves of Fig. 5b,c. We observe that the capacitance increases progressively on structuring with rods, sheets and the maximum being for the sheets-rods hierarchical assembly compared to a planar substrate. The Ragone plots of Fig. 5d also show that the energy density of the hierarchical structure is improved by an order of magnitude over planar capacitor. The complete structure of the capacitor is made by electrochemical deposition without the use of any patterning process or the use of metal foam.

In summary, high density hierarchical structures without any underlying pattern are used for making an interlocked, high surface area tactile device. The morphology of the structure consisting of interpenetrating ZnO 2-D sheets with 1-D rods on them serving as interconnects leads to a highly sensitive and dynamic modulation of the conductive pathways with pressure. While structuring with a single nanostructure leads to improvement in device performance, combining multiple structures in a rational way leads a significant enhancement in the device properties. The temperature sensitivity of the device is based on the properties of ZnO. The ability to sense both pressure and temperature in a single device structure with a simple fabrication procedure will bring the electronic tactile devices closer to skins ability and at the same time make them easily accessible. The hierarchical structure presented here can also be of significance for applications in adhesives, hydrophobic surfaces and also sensing of shear stresses. Other rational combinations of nanomaterials such as 0-D on 2-D sheets or use of other morphologies such as nano-prisms can further lead to enhancement of specific properties such as array of electrodes with high electric fields^55^ which will be of significance for application in electrochemical devices and sensing.

## Methods

### Hierarchical electrochemical deposition of ZnO nanostructures and MnO_2_

ITO coated glasses with a sheet resistance of 10 ohms obtained from Delta Technologies were used as substrates for the hierarchical growth of ZnO nanostructures. The electrochemical deposition was carried out by using an Ivium CompactStat Electrochemical Analyser with a standard three-electrode setup. A Ag/AgCl electrode was used as the reference electrode, with a platinum wire as the counter electrode for all depositions. Different substrates were connected to the working electrode during each step of the deposition. For the ZnO nanosheets, a clean ITO substrate was used as the working electrode. A constant electric potential of −1.0 V was applied for one hour. 0.028 M ZnCl_2_ with 0.1 M KCl aqueous solution used as the electrolyte. A beaker filled with the electrolyte solution was immersed in a silicon oil bath, which was heated on a hot plate, to maintain a steady temperature of 75 °C during the deposition. Substrates were washed thoroughly with Millipore water after the deposition and dried under a nitrogen stream. The substrates were then heated at 350 °C for one hour for the annealing of ZnO nanosheets. A thin layer of gold (~15 nm) was coated on ZnO nanosheets using magnetron sputtering to improve the charge collection capacity. For deposition of nanorods, the substrate was again used as the working electrode. Same voltage of −1.0 V was applied for the same amount of time at the same temperature as in the first step. A lower concentration of 1.25 mM ZnCl_2_ with 0.1 M KCl aqueous solution was used as the electrolyte^48^. Same cleaning and annealing process was followed. To electrochemically deposit a film of MnO_2_ on ZnO nanostructure template, a thin layer of gold was sputtered again on the substrate that has ZnO nanostructure. The substrate was immersed in a plating solution of 20 mM Mn(NO_3_)_2_ with 100 mM NaNO_3_. A constant current of 100 μA cm^−2^ was applied for 5 minutes^56^. The substrate was washed with Millipore water after the deposition to remove excessive electrolyte and then annealed on a hot plate at 60 °C for one hour.

### Characterizations

Zeiss Ultraplus field emission scanning electron microscopy (FESEM) equipped with energy-dispersive X-ray spectroscopy (EDX) was used to examine the morphologies of hierarchical ZnO structure and MnO_2_ nanoparticles. The crystal structures were characterized by glancing incidence X-ray diffraction (GIXRD) using a PANalytical X’Pert Pro MRD diffractometer with Cu Kα radiation (λ = 1.54 Å) at an incidence angle of 0.4°. A Keysight 3458 A Digital Multimeter and a Keysight 6614 C 50 Watt System Power Supply were used for measuring current responses and applying the potential bias, respectively. Our samples were connected in series with the multimeter and power supply to form a circuit. A two probe method was used by connecting one probe to the ITO layer on the bottom sample directly and connecting the other probe to the ITO substrate on the top sample through a very small amount of Gallium-Indium eutectic. By using the highly conductive liquid metal alloy, we could maintain a good electric contact with minimum pressure. An MFA Motorized Miniature Linear Stage was used to apply the load to the samples. A Honeywell Model 31 miniature load cell was connected to the stage for measuring the load. Combined with a Micro-pro series digital panel meter, we were able to read out the measurements and record the data. For the temperature sensitivity test, the temperature of the samples was controlled by changing the temperature setting on a hot plate which the samples were sitting on. Accurate temperature readings on the sample surface were measured by an infrared thermometer. Electrochemical characterizations were done again by using the Ivium CompactStat Electrochemical Analyser. For cyclic voltammetry and galvanostatic charge/discharge, two symmetric samples were separated by a porous ion-permeable membrane (separator) as working and counter electrodes, with a Ag/AgCl electrode as the reference electrode.

## Electronic supplementary material

Supplementary Information
