# Supplementary material for: Bio-inspired interlocking random 3-D structures for tactile and thermal sensing
Source: Sci Rep. 2017 Jul 19;7:5834. doi: 10.1038/s41598-017-05743-w (PMC5517528; doi:10.1038/s41598-017-05743-w)
Supplement: Supplementary file 1 — Supplementary Information [file 41598_2017_5743_MOESM1_ESM.pdf]

Supporting Information

Bio-inspired interlocking random 3-D structures for tactile and thermal sensing

*Long Pu, Rohit Saraf, and Vivek Maheshwari**

Department of Chemistry, Waterloo Institute of Nanotechnology, University of Waterloo, Waterloo, ON N2L 3G1, Canada

Corresponding Author: *E-mail: vmaheshw@uwaterloo.ca

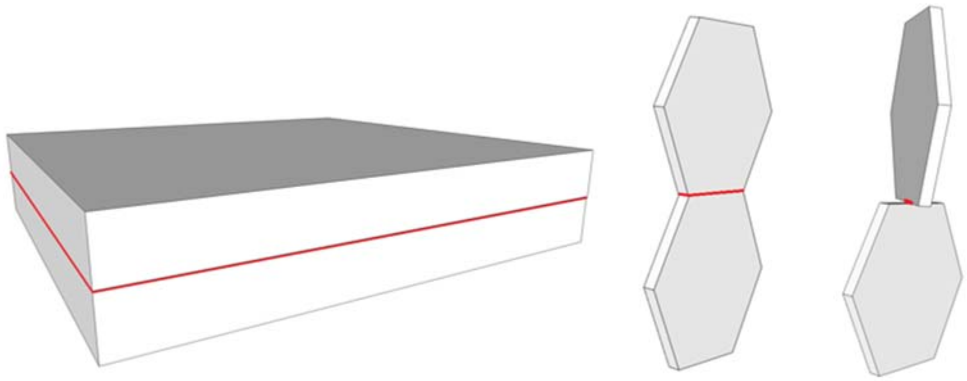

Figure S1. A schematic of how the ZnO structure intensifies the applied pressure at the contact points (contact area is noted by the red line).

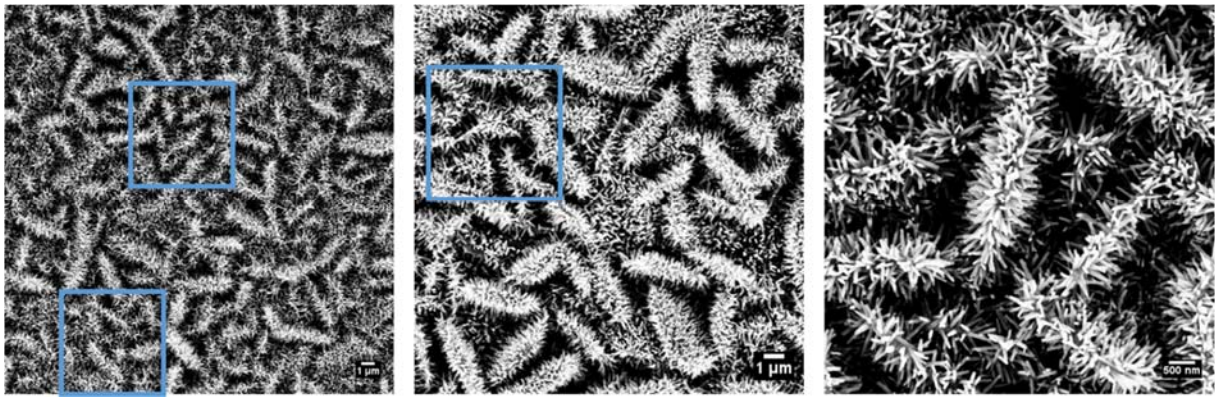

Figure S2. FESEM images of ZnO nanostructure at different magnifications (5 µm by 5 µm areas are denoted in blue squares).

By analyzing the FESEM images, the average diameter of ZnO nanosheets was measured to be around 3 µm with an average thickness of around 100 nm. In a typical 5 µm by 5 µm area, there are approximately 9-12 nanosheets which cover 10.8-14.4% of the total area. The decrease in contact area will increase the pressure experienced by the sheets edges by 7-9 times. The random orientation of nanosheets also plays an important role by further decreasing the contact area to a very small fraction on the edge, eventually increasing the effective pressure.

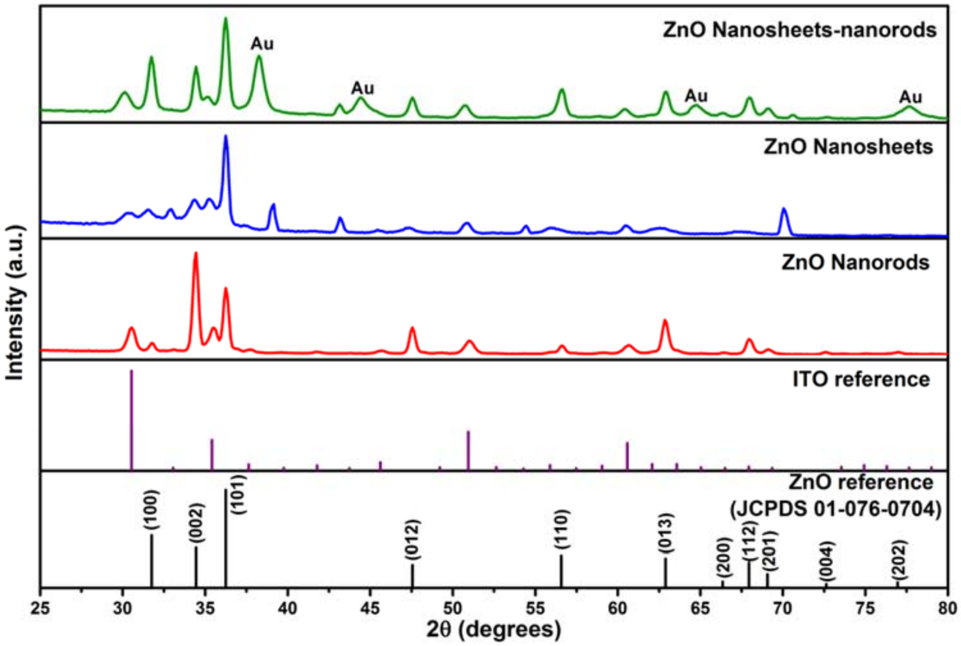

Figure S3. GIXRD patterns of ZnO nanorods, nanosheets, and nanosheets-nanorods, along with the standard ITO glass substrate and a powder ZnO reference sample.

Fig. S3 shows the intense XRD peaks from the ZnO and the weaker diffraction features due to the ITO substrate. These XRD features are consistent with the hexagonal wurzite structure in the reference pattern for ZnO powder (JCPDS 01-076-0704). In particular, the features at 31.7°, 34.4°, and 36.2° are assigned, respectively, to the ZnO (100), (002), and (101) planes, consistent with the most prominent XRD features in the reference powder spectrum. Among the three aforementioned ZnO features, the diffraction in (002) [or (0001)] plane is strongest for the nanorods, while the (101) [or ($10\bar{1}0$)] plane is dominating for the nanosheets. The diffraction features from the (100), (002), and (101) planes indicating the preferential growth directions of ZnO nanosheets-nanorods is observed in the respective spectrums.

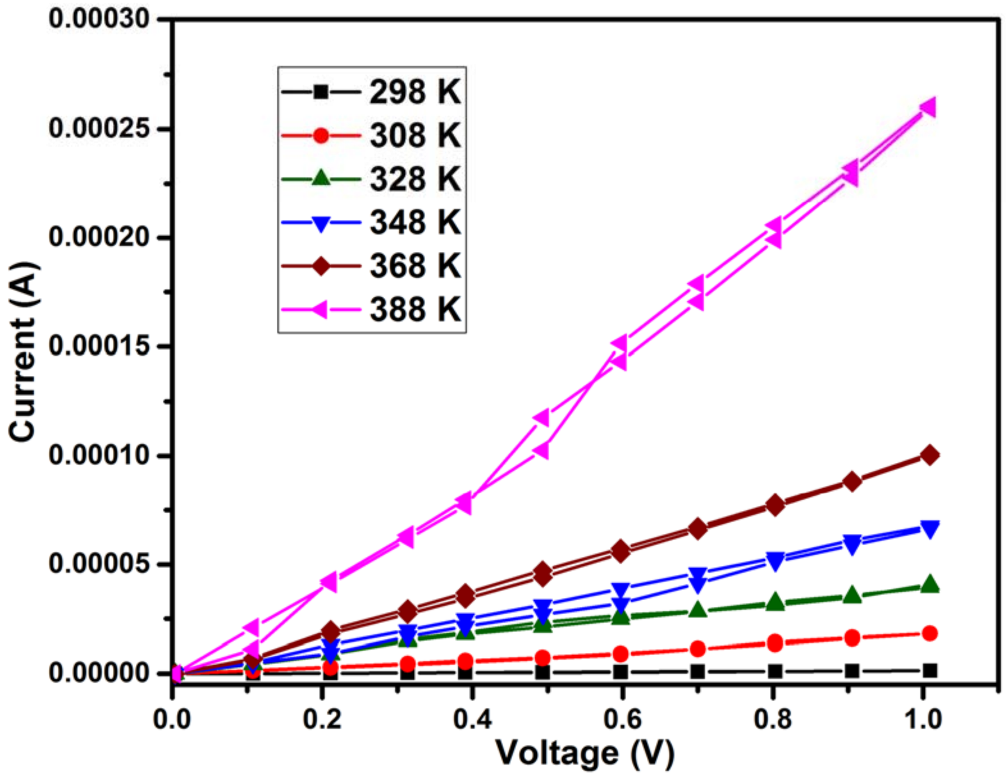

Figure S4. Current-voltage curves of ZnO hierarchical sensor at different temperatures.

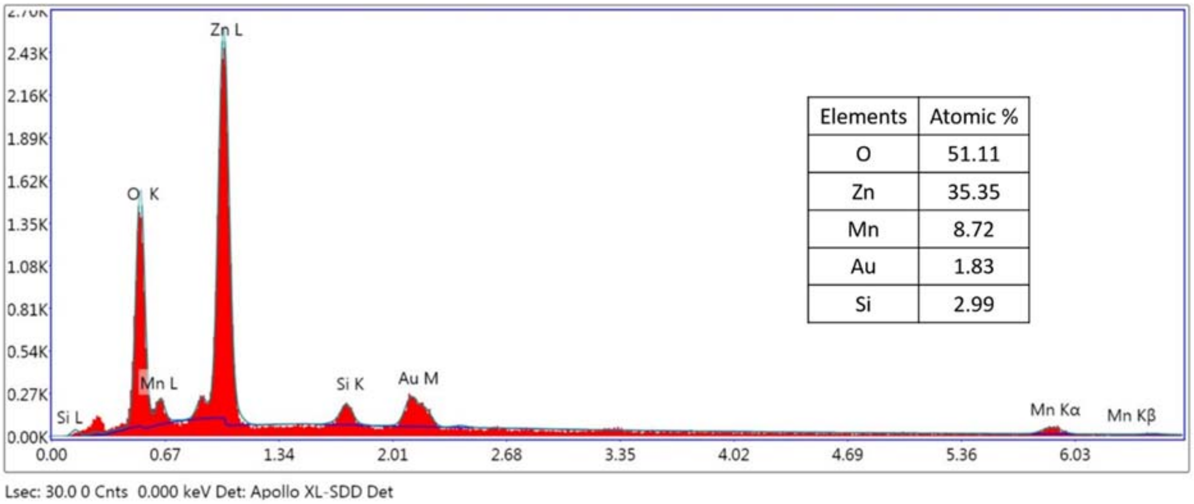

Figure S5. EDX spectrum and elemental composition of electrochemically deposited MnO_2 layer on ZnO structure.

EDX spectrum shows the presence of Zn, O, Mn, Au, and Si elements, the Si peak is due to the silicon detector. The sample possesses good stoichiometry in agreement with the chemical composition of the respective elements.
